# Esters of levonorgestrel and etonogestrel intended as single, subcutaneous-injection, long-lasting contraceptives

**DOI:** 10.1016/j.steroids.2018.07.010

**Published:** 2018-09

**Authors:** Frederick A. Meece, Gulzar Ahmed, Hareesh Nair, Bindu Santhamma, Rajeshwar R. Tekmal, Chumang Zhao, Nicole E. Pollok, Julia Lara, Ze'ev Shaked, Klaus Nickisch

**Affiliations:** a Evestra, Inc, 6410 Tri County Pkwy, Schertz, TX 78154, United States; b University of Texas Health Science Center San Antonio, 7703 Floyd Curl Dr., San Antonio, TX 78229, United States

**Keywords:** Injectable contraception, MPA, Levonorgestrel, Etonogestrel, Prodrug

## Abstract

An effort with the goal of discovering single-dose, long-lasting (>6 months) injectable contraceptives began using levonorgestrel (LNG)-17-β esters linked to a sulfonamide function purposed as human carbonic anhydrase II (hCA 2) ligands. One single analog from this first series showed noticeably superior anti-ovulatory activity in murine models, and a subsequent structure-activity relationship (SAR, the relationship between a compound’s molecular structure and its biological activity) study based on this compound identified a LNG-phenoxyacetic acid ester analog exhibiting longer anti-ovulatory properties using the murine model at 2 and 4 mg dose than medroxyprogesterone acetate (MPA). The same ester function linked to etonogestrel (ENG) furnished a compound which inhibited ovulation at 2 mg for 60 days, the longest duration of all compounds tested at these doses. By comparison, MPA at the same dose inhibited ovulation for 32 days.

## Highlights

• Carbodiimide-mediated esterification of levonogestrel and etonogestrel.
• First examples of sulfonamide-derived norgestrel analogs.
• Two compounds identified as effective anti-ovulation agents in the murine model.

## Graphical abstract

**Figure:**
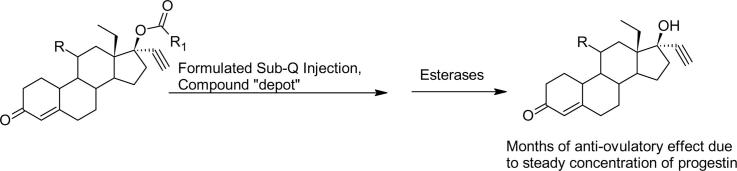

## 1 Introduction

**Fig. 1:**
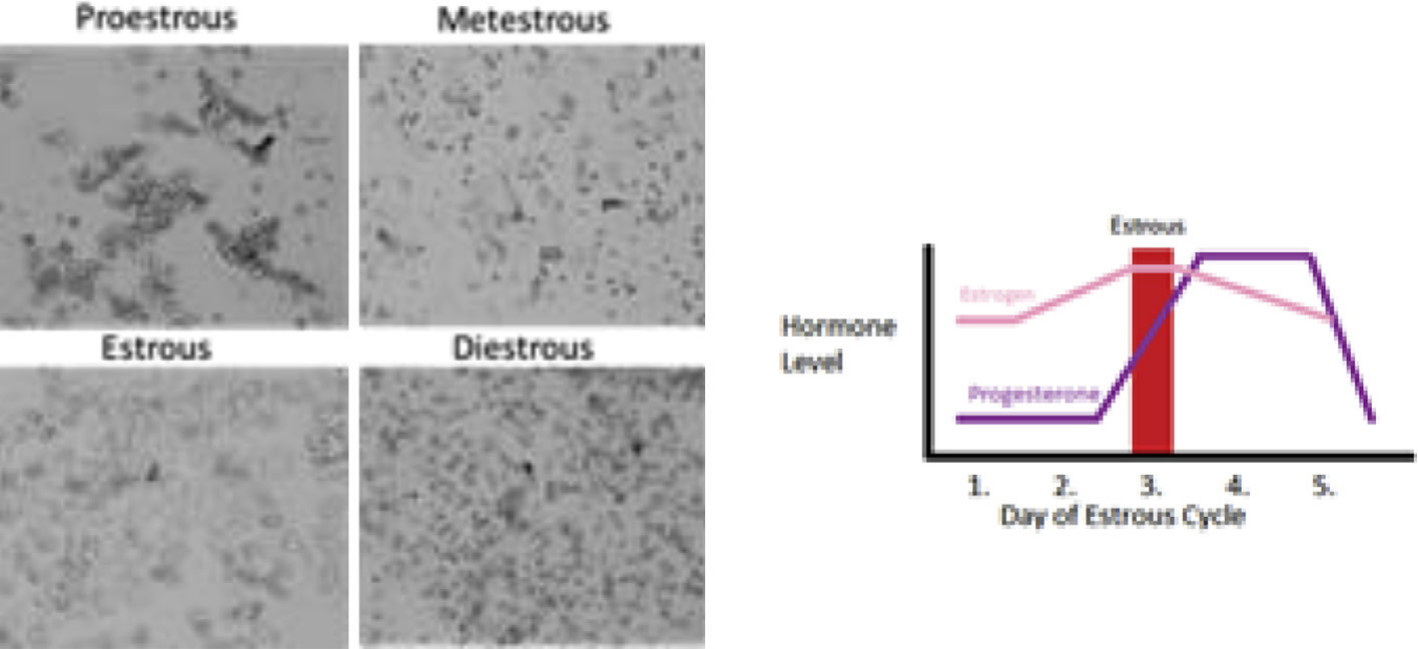
Left-microscopic appearance of murine vaginal smears at each stage of the estrous cycle. Right-description of estrogen and progesterone levels through the estrous cycle.

As of 2011, forty million women worldwide used injectable contraception [1] which is a preferred delivery system of contraceptive agents especially in southeastern Asian countries such as Thailand, the Philippines, and sub-Saharan Africa. While the method remains popular with users, one obstacle to regular use is availability, due to regional product unavailability and/or no clinical access, among other reasons [2]. The idea of injectable contraception lasting over six months which reduces side effects and improves safety profiles could in theory prove valuable in negating the availability issues, while benefiting women and health care systems globally (see Fig. 1).

MPA a widely used injectable contraceptive, and is commercially available as two different products- one is a intramuscular injection containing 150 mg of MPA and the other a sub-cutaneous injection containing 104 mg of MPA. Depo-Sub Q Provera 104 is a sub-cutaneous single-injectable formulation of the progestin medroxyprogesterone acetate (MPA) which among other uses, provides a contraceptive effect for 13 weeks post injection by preventing ovulation at a typical failure rate of 3% [3], [4]. The approved dose for Depo Provera is 150 mg in 1 ml for intramuscular injection and 104 mg in 0.65 ml for sub-cutaneous injection-formulation [5]. There are benefits from a solely progestin contraceptive product like Depo-Provera, including no increased risk of deep vein thrombosis (DVT), pulmonary embolism (PE), stroke, or myocardial infarction as with estrogen-based contraceptives, and a decreased rate of endometrial cancer and a decrease of symptoms associated with endometriosis [6]. There are side effects caused by MPA, including weight gain, mood swings, acne and hirsutism. In 2004, a black box warning was issued by the United States Food and Drug Administration indicating loss of bone mineral density and recommended that patients be prescribed the drug for no longer than two years due to this risk [7].

Levonorgestrel (LNG) is currently a very commonly prescribed progestin used in contraception. Most of the reported side effects of MPA are not (or to a significantly lower extent) observed in women taking LNG. A longer acting injectable contraceptive product based on LNG or ENG could provide greater convenience for women who seek injectable contraception. Risks of progestin-only contraception include potential problems with bleeding, possibly inducing amenorrhea. This might be curtailed by a rhythmic and steady, low-dose of progestin which would still enable contraception, provided by the single-dose, long-lasting depot of properly formulated progestin. It might therefore be very desirable to develop a long-term injectable contraceptive product based on LNG [8].

In 1975, the World Health Organization set forth an initiative to find novel, long-lasting contraceptive agents. Several groups discovered and published findings involving long-lasting contraceptive agents which were in essence 17-β esters of LNG and to a lesser extent, norethisterone [9], [10], [11]. These esters were suspended in various media and then introduced into rats via sub-cutaneous injections. One in particular showed superior anti-ovulation activity in murine models, namely the butyrate ester of LNG known as levonorgestrel butyrate (LB). This compound inhibited ovulation in the murine model for 32 days at a 2 mg dose [10]. LB underwent several clinical trials but to this date has not been marketed [12]. A more recent study [13] found that a higher body mass index of women resulted in earlier return to ovulation, suggesting possible inconsistencies in efficacy from woman to woman.

Recently, our group published work regarding steroidal esters linked to sulfonamides through amino acid linkers to improve oral bioavailability and to prevent first-pass liver metabolism [14]. These pro-drugs act as hCA 2 ligands and accumulate in red blood cells, which in effect protects the compounds from being metabolized by the liver. Theoretically this has two main positive effects, one in being that this makes the steroids safer by curtailing liver toxicity from metabolism and the other being a marked improvement in oral bioavailability as the compound will be delivered straight to the vasculature upon intestinal absorption. Cleavage of the ester by endogenous esterases then frees the active molecule to then travel on to its respective target. A hypothesis was formed involving this same concept being applied then to sub-cutaneous injections of sulfonamide-linked esters of LNG; from the injection site slow release of prodrug into sub-cutaneous tissue followed by uptake into capillaries and erythrocytes laden with hCA 2 would result in ideal systemic concentration, assuming a steady state over time. Herein, we report our work and the results regarding ovulation inhibition by a series of LNG esters and one ENG ester.

## 2 Experimental methods

### 2.1 General methods

Nuclear magnetic resonance (NMR) spectra were recorded on a Bruker ARX (300 MHz) spectrometer as deuterochloroform (CDCl_3_) or DMSO‑*d*_6_ solutions using tetramethylsilane (TMS) as an internal standard (ppm = 0) unless noted otherwise. High-performance liquid chromatography (HPLC) was performed on a Waters Alliance 2695 with a photodiode array 2996 system using a Waters XTerra® RP18 column (3.5 µm, 4.6 × 150 mm). All compounds used in anti-ovulation studies had purity than >95% at MAX plot as determined by this system. Medium-pressure liquid chromatography (MPLC) was performed using a Isolera One from Biotage USA® (Charlotte, NC) utilizing SiliaFlash® P60 40–63 µm silica gel from Silicycle (Quebec, Ontario). Thin-layer chromatography (TLC) analysis were carried out on silica gel GF (Analtech) glass plates (2.5 cm × 10 cm with 250 µm layer, pre-scored). Most chemicals and solvents were analytical grade and used without further purification unless otherwise noted. LNG was supplied as a gift from Wyeth (DE) and ENG was purchased from Nexconn (PRC). A Mini-Mill Pulverisette 23 purchased from Fritsch was used to mill the solid compounds for formulation. Female Sprague-Dawley rats were purchased from Charles River (Wilmington, MA). Clean tip sponge swabs were purchased from Texwipe (Kernsville, NC), and vaginal smears were visualized using a Nikon Eclipse Ti-S microscope.

### 2.2 Chemistry

**3** (bold numbers designate experimental compounds). (13S,17R)-13-ethyl-17-ethynyl-3-oxo-2,3,6,7,8,9,10,11,12, 13,14,15,16,17-tetradecahydro-1H-cyclopenta[a]phenanthren-17-yl(3-sulfamoylphenoxy)acetate (General method #1): To an oven-dried round bottom flask was added **2** (973 mg, 2.5 mmol), 3-hydroxybenzenesulfonamide, and 4 Å molecular sieve. Anhydrous DMF was added (5 ml) and then cesium carbonate (814 mg, 2.5 mmol). The mixture was allowed to stir for 24 h, after which time an additional 0.5 equivalent of base and 0.3 equivalent of phenol was added to fully convert the starting material by allowing to stir an additional 16 h. The mixture was then diluted with ice-cold saturated sodium bicarbonate and the resulting solids collected by vacuum filtration, washed with water and allowed to air-dry. The product was isolated by subjecting the crude solid to flash chromatography using a 2–10% gradient of acetone in DCM. ^1^H NMR (300 MHz, DMSO‑*d*_6_) *δ* 7.52–7.31 (m, 4H), 7.13 (ddd, *J* = 7.8, 2.4, 0.9 Hz, 1H), 5.73 (s, 1H), 4.83 (s, 2H), 3.65 (s, 1H). IR (cm^−1^): 3324, 3294, 3231, 2934, 2866, 1758, 1742, 1658, 1343, 1209, 1158, 793, 680. MP 166.5–169 °C.

**5**. (13S,17R)-13-ethyl-17-ethynyl-17-hydroxy-2,7,8,9,10,11,12, 13,14,15,16,17-dodecahydro-1H-cyclopenta[a]phenanthren-3-yl 4-sulfamoylbenzoate. To a round bottom flask was added **1** (2.4 g, 7.7 mmol) and cesium carbonate (3.0 g, 9.2 mmol). Anhydrous toluene (100 ml) was added, and a Dean-Stark trap was placed atop the flask and 75 ml of toluene was removed from the trap. 4-Sulfamoylbenzoyl chloride was then added (2.0 g, 9.2 mmol) and the mixture was allowed to reflux for 24 h, upon which time the mixture was allowed to cool and then washed with 2 M HCl. The layers were separated and extracted with ethyl acetate. The combined organic layers were washed with saturated sodium bicarbonate, brine, and dried over sodium sulfate. The crude was then subjected to flash chromatography using 10% acetone in DCM. A further reverse-phase medium pressure liquid chromatography procedure was used (45–55% gradient of acetonitrile) to afford the purified product as a foam; ^1^H NMR (300 MHz, DMSO‑*d*_6_) *δ* 8.18 (d, *J* = 8.7 Hz, 2H), 7.97 (d, *J* = 8.7 Hz, 2H), 7.58 (s, 2H), 5.96 (s, 1H), 5.54 (bs, 1H), 5.29 (s, 1H), 0.919 (t, *J* = 6.9 Hz, 1H).

**17**. (13S,17R)-13-ethyl-17-ethynyl-3-oxo-2,3,6,7,8,9,10,11,12, 13,14,15,16,17-tetradecahydro-1H-cyclopenta[a]phenanthren-17-yl 4-sulfamoylbenzoate. Formation of 4-{bis[(2,4-dimethoxy-phenyl)methyl]sulfamoyl}benzoic acid: A round bottom flask was charged with 4.87 g (15.5 mmol) of bis(2,4-dimethoxybenzyl)amine and TEA (3.9 ml, 28.1 mmol) and dissolved in THF (30 ml) and chilled to 0 °C. Then methyl 4-(chlorosulfonyl)benzoate (3.3 g, 14.1 mmol) in 45 ml THF was added dropwise to the amine solution. The mixture was then allowed to gradually warm to room temperature while stirring overnight. The next day the mixture was evaporated onto silica and subjected to flash chromatography using a 5–20% gradient of EtOAc in DCM to obtain 5.94 g (82%) of the intermediate 4-{bis[(2,4-dimethoxyphenyl)methyl]sulfamoyl}benzoic acid methyl ester; ^1^H NMR (300 MHz, CDCl_3_) 8.03 (d, *J* = 8.9 Hz, 2H), 7.70 (d, *J* = 8.5 Hz, 2H), 7.15 (d, *J* = 8.9 Hz, 2H), 6.38 (dd, *J* = 8.4, 2.6 Hz, 2H), 6.25 (d, *J* = 1.2 Hz, 2H), 4.41 (s, 4H), 3.96 (s, 3H), 3.78 (s, 6H), 3.59 (s, 6H). The methyl ester was saponified by treating with 23 ml of 5 M NaOH in 100 ml THF at reflux for 4 h to afford the acid (isolated by removal of THF, then treating with sodium hydrogen sulfate until pH was 3 and extracting with ethyl acetate (5.77 g recovered, 99%). 3.28 g (6.5 mmol) of this material was then placed into a round bottom flask along with **1** (511 mg, 1.64 mmol) and DMAP (0.2 g, 1.64 mmol). The materials were dissolved in DCM (15 ml) and then DIC added (1.0 ml, 6.5 mmol) once a homogenous solution was observed and the mixture was allowed to stir overnight. The next day the solids were removed via filtration and the crude material subjected to flash chromatography using 3–5% acetone in DCM to give 1.16 g of the intermediate (89%). This was then suspended in DCM (11 ml) and chilled to 0 °C before addition of TFA (5.5 ml) and allowed to stir one hour at which time TLC and HPLC indicated the conversion complete. The volatiles were then removed under vacuum and the solid residue washed with saturated sodium bicarbonate. The crude solids were then subjected to flash chromatography using 5–10% acetone in DCM gradient to afford the title compound in 72% yield; ^1^H NMR (300 MHz, DMSO‑*d*_6_) *δ* 8.08 (d, *J* = 7.8 Hz, 2H), 7.96 (d, *J* = 7.5 Hz, 2H), 7.58 (s, 2H), 5.74 (s, 1H), 3.70 (s, 1H). IR (cm^−1^): 3264, 2940, 2873, 1723, 1651, 1341, 1273, 1166, 1091, 767. MP 173.5–178 °C.

**18**. (13S,17R)-13-ethyl-17-ethynyl-3-oxo-2,3,6,7,8,9,10,11,12, 13,14,15,16,17-tetradecahydro-1H-cyclopenta[a]phenanthren-17-yl sulfamoylacetate.

Formation of {bis[(2,4-dimethoxyphenyl)methyl]sulfamoyl}-acetic acid-A round bottom flask was charged with chloro-sulfonylacetyl chloride (1.2 ml, 11.3 mmol), dissolved in DCM (40 ml) and then anhydrous methanol was added (0.46 ml, 11.3 mmol) and allowed to stir for 1 h. Then 3.74 g (11.9 mmol) of bis(2,4-dimethoxybenzyl)amine and TEA (3.15 ml, 22.6 mmol) were added to the reaction mixture and allowed to stir overnight while gradually warming to room temperature, following which time the mixture was rotovapped onto silica and subjected to flash chromatography using ethyl acetate (0–8%) in DCM to afford the intermediate as the methyl ester (1.95 g, 38% yield). The ester was saponified using LiOH-H_2_O (5 M solution, ten equivalents) in THF at room temp, then the THF removed, and the residue treated with sodium hydrogen sulfate to neutralize and extracted into ethyl acetate, giving the acid in quantitative yield; ^1^H NMR (300 MHz, DMSO‑*d*_6_) 7.04 (d, *J* = 8.6 Hz, 2H), 6.50–6.43 (m, 4H), 4.25 (s, 4H), 4.07 (s, 2H), 3.74 (s, 6H), 3.70 (s, 6H). 1.95 g (4.3 mmol) of this material was then placed into a round bottom flask along with **1** (336 mg, 1.08 mmol) and DMAP (0.131 g, 1.08 mmol). The materials were dissolved in DCM (15 ml) and then DIC added (0.66 ml, 4.3 mmol) once a homogenous solution was observed and the mixture allowed to stir overnight. The next day the solids were removed via filtration and the crude material subjected to flash chromatography using 3–5% acetone in DCM. The intermediate was then suspended in DCM, (2 ml) and chilled to 0 °C before addition of TFA (1.0 ml) and allowed to stir one hour at which time TLC and HPLC indicated the conversion complete. The volatiles were then removed under vacuum and the solid residue washed with saturated sodium bicarbonate. The crude solids were then subjected to flash chromatography using 5–10% acetone in DCM gradient to afford the title compound in 94% yield; ^1^H NMR (300 MHz, DMSO‑*d*_6_) *δ* 7.41 (s, 2H), 5.73 (s, 1H), 4.06 (s, 2H), 3.65 (s, 1H). IR (cm^−1^): 3278, 2940, 2886, 1739, 1651, 1348, 1166, 1112, 653. MP 148.5–152 °C.

**20**. (13S,17R)-13-ethyl-17-ethynyl-3-oxo-2,3,6,7,8,9,10,11,12,13,14,15,16,17-tetradecahydro-1H-cyclopenta[a]phenanthren-17-yl 4-sulfamoylbenzoate, sodium. Compound **18** (0.3 g, 0.60 mmol) was dissolved in THF (5 ml) and chilled to 0 °C. The mixture was then treated with 2 M NaOH and stirred vigorously for 5 min, then the mixture was transferred to a separatory funnel and the layers separated, the aqueous was extracted with ethyl acetate. The combined extracts were concentrated to reveal a yellowish solid (233 mg); ^1^H NMR (300 MHz, DMSO‑*d*_6_) *δ* 7.84 (d, *J* = 8.1 Hz, 2H), 7.76 (d, *J* = 8.1 Hz, 2H), 5.75 (s, 1H), 3.65 (s, 1H). IR (cm^−1^): 3441, 3282, 2938, 2866, 1725, 1660, 1272, 1255, 1080, 965, 768. MP >315 °C.

**25**. (13S,17R)-13-ethyl-17-ethynyl-3-oxo-2,3,6,7,8,9,10,11,12,13,14,15,16,17-tetradecahydro-1H-cyclopenta[a]phenanthren-17-yl (3-methylphenoxy)acetate (General Method #2): To a round bottom flask was added **1** (1.41 g, 4.5 mmol), (3-methyl-phenoxy)acetic acid (3.0 g, 18.1 mmol), and DMAP (550 mg, 4.5 mmol) in DCM (30 ml) at ambient temperature. Once a homogenous solution was observed, DIC was added (2.8 ml, 18.1 mmol). The mixture was allowed to stir overnight. The next morning the mixture was filtered, and the filtrate was rotovaped onto silica gel and subjected to flash chromatography. The resultant foam was then crystallized from methanol and DCM yielding 1.08 g of white crystal (73%). ^1^H NMR (300 MHz, CDCl_3_) *δ* 7.17 (t, *J* = 7.5 Hz, 1H), 6.81 (d, *J* = 7.2 Hz, 1H), 6.73–6.67 (m, 2H), 5.84 (s, 1H), 4.59 (s, 2H), 2.66 (s, 1H), 2.33 (s, 3H). IR (cm^−1^): 3227, 1775, 1658, 1611, 1259, 1196, 1158, 1095, 785, 688. MP 187–190 °C.

### 2.3 Formulation

Vehicle-

**Table:** 

Component	Amount
Benzyl alcohol	1 g
Methyl cellulose	1 g
Sodium phosphate dibasic dihydrate	0.752 g
Sodium phosphate monobasic dihydrate	2.99 g
Deionized water	200 ml

All solids added to water and mixed under stirring for 24 h at ambient temperature.

Mill: Fritsch pulverisette 23.

#### 2.3.1. Original formulation

A stainless steel grinding bowl with a lid and seal along with three 10 mm stainless steel balls were used to formulate suspensions for injection. The compounds were weighed and added to the grinding bowl and steel balls. 1.5 ml of formulation vehicle was added to the grinding bowl, via pipette. The milling conditions used as listed: 20 min, 20 Hz. After milling, the suspension was transferred to a volumetric flask. Formulation vehicle was used to dilute the milled suspension to produce the desired injection dose.

### 2.4 Biology

Long acting properties of each preparation had been determined in an estrous suppression assay using virgin, mature (180–200 g) cycling rats of Sprague-Dawley strain. Upon receipt the animals were smeared daily (procedure described as below) and those exhibiting two consecutive cycles were used for the study. Each animal was injected subcutaneously (s.c) with 0.5 ml of the test preparation (in the vehicle as described above) on the same day regardless of the stage of the cycle. Each compound was initially tested in 6 rats. Every other day excluding weekend, smears were taken starting on the day after injection and continued until such time that cornification of vaginal epithelium was observed and cycling was re-established [10].

## 3 Results and discussion

### 3.1 Chemistry

**Scheme 1:**
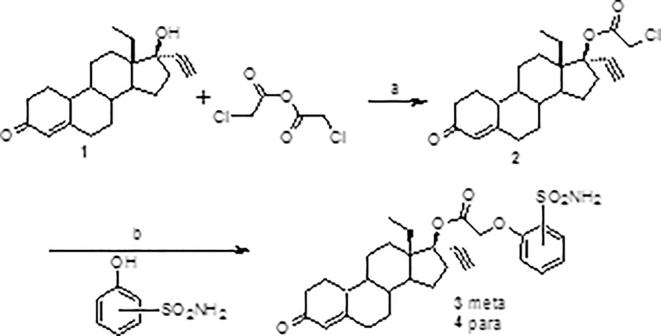
Synthesis of LNG-sulfamoylphenoxyacetic acid esters: (a) DMAP, toluene, reflux, 16 h; (b) Cs_2_CO_3_, DMF, rt.

**Scheme 2:**
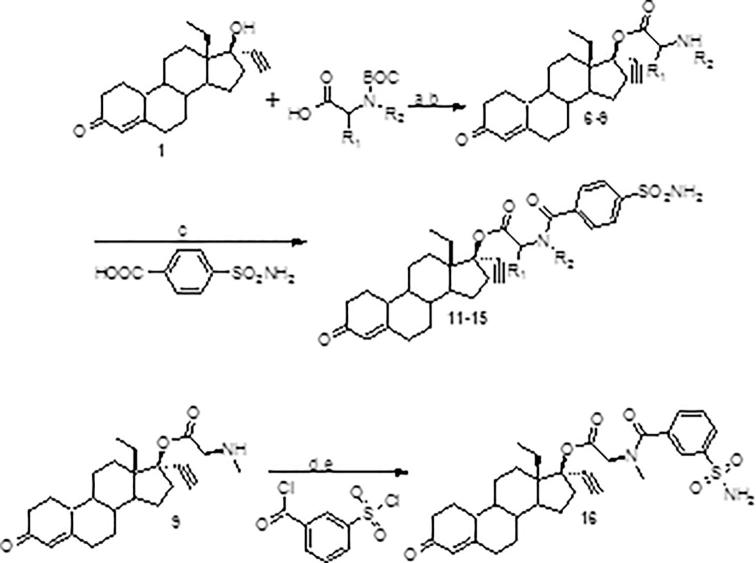
Synthesis of phenylsulfamoyl amino acid linked LNG esters: (a) DIC/DMAP/DCM, rt; (b) conc HCl/EtOAc; (c) DIC/HOBt/DCM, rt; (d) DIEA/THF, then NH_4_OH (e).

**Scheme 3:**
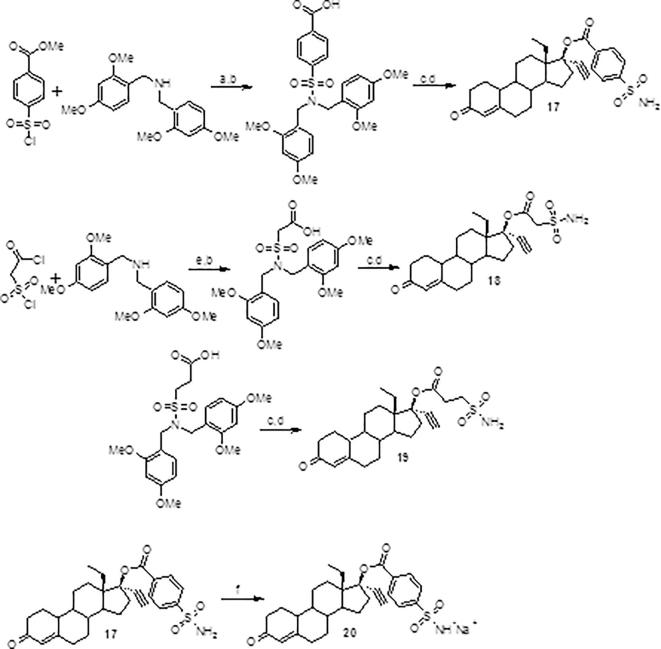
Synthesis of aryl and aliphatic sulfamoyl LNG esters: (a) TEA/THF or DCM, 0 °C to rt; (b) NaOH/THF, reflux; (c) **1**./DIC/DMAP/DCM, rt; (d) TFA/DCM, 0 °C; (e) MeOH/TEA/DCM, −78 °C to rt; (f) 2 M NaOH/THF, rt.

An initial series of compounds were synthesized as shown in Scheme 1, Scheme 2, Scheme 3 and Eq. 1 below.

The alpha-chloro LNG intermediate **2** (Scheme 1) was formed in moderate yields (5 equivalents of anhydride, 1 equivalent of DMAP) and then the chloride displaced by the cesium phenolate in DMF. The 3-hydroxybenzenesulfonamide reagent was synthesized as described previously [15].

**Figure:**
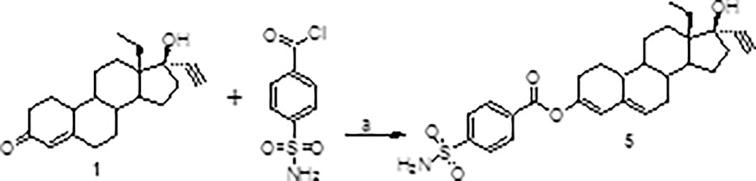

*Eq. 1*. Synthesis of 3-enol-sulfamoylaryl esters: (a) Cs_2_CO_3_, toluene or Tl_2_CO_3_, dibenzo-18-crown-6, toluene, reflux.

This analog (**5**, Eq. 1) was the main product formed using conditions described in previous articles [9], [16] regarding the use of group I metal carbonate salts to form 17-β esters of LNG and norethisterone with acid chlorides utilized as the acylating agents. There are several possible explanations for the regiochemistry being different in this case. As the acid chlorides being used in these earlier articles are simple, aliphatic species as opposed to aromatic sulfonamides, it is possible that under these conditions acylation of the enol tautomer is prevalent rather than at 17-OH by the acid chloride. Another possibility is that the authors were mistaken in assigning the structure of their acylation products. As no NMR spectral data is included in these previous articles, this possibility cannot be verified without repeating their procedures using the aliphatic acid chlorides. In addition to analog 5 a 4,4′-biphenylsulfonamide analog was made in the same manner.

As noted earlier, our previous work focused on steroid-sulfonamide conjugates linked using amino acids, so a large portion of our first series (Scheme 2, Table 1) also made use of this same motif. Previously described methods of forming aliphatic esters through mixed anhydride formation [10], [17] utilizing trifluoroacetic anhydride were either completely ineffective or created a complex, intractable mixture of products when using N-protected amino acids as substrates. Classic Steglich-type esterification at first also proved ineffective, despite using an excess of amino acid, and catalytic amounts of DMAP. After screening various parameters of the esterification procedure, it was found that by using five equivalents of acid (this was later pared back to four) and one equivalent of DMAP, the esterification proceeded well and good to excellent yields were observed. One crucial aspect to the success of this acylation was that the carbodiimide (we mainly used N,N′-diisopropylcarbodiimide) be added last to the mixture. The reaction concentration was also important to achieving the excellent yields, with a requirement that at least 0.1 M with respect to the steroid be maintained. The protecting group was then removed and then amide formation using sulfamoylbenzoic acid and DIC and HOBt was performed to afford the analogs. We also used other sulfamoyl acids to form amides, but as these were not tested in our anti-ovulation assay, they have been omitted from this article.

**Table 1:** Compounds **6**–**9,15**.

Compound	R_1_	R_2_
**6**	R_1_-R_2_-propyl	–
**7**	Me	H
**8**	Me	Me
**9**	H	Me
**15**^a^		

a Azetidine beta amino acid.

The conditions used to successfully form amino-acid esters of LNG described in Scheme 2 did not work when either an alkyl or aryl sulfamoyl carboxylic acid was the substrate. This was later attributed to the presence of the sulfonamide group. Masking the acidic protons by making use of the bis-2,4-dimethoxy-benzylamino blocking group [18] (Scheme 3) allowed for the successful employment of the conditions described in Scheme 2 and good to excellent yields were once again observed. The protecting group was removed in the final step by treatment with TFA. It is noted that the sulfonyl chloride carboxylic acid was protected as its methyl ester prior to chloride displacement with bis-2,4-dimethoxybenzylamine. This allowed for much better yields and ease of isolation. Once the conjugate was in hand, the carboxylic acid was easily attainable (work up and isolation made use of either potassium or sodium hydrogen sulfate to bring the pH down to about 2–3, so as not to remove the bis-2,4-dimethoxyamino group) and then used in the subsequent carbodiimide mediated esterification of LNG.

**Figure:**
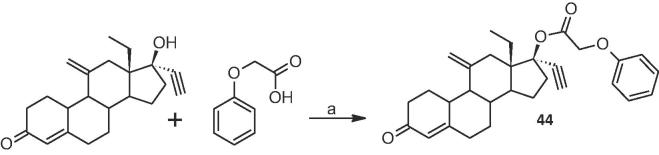

*Eq. 2.* Synthesis of phenoxyacetic acid ester of ENG: (a) DIC/DMAP/DCM, rt.

Ester formation minus the sulfonamide function (Table 2) proceeded in either one of two routes-displacement of the alpha chloride from intermediate **2** using cesium carbonate to generate the cesium phenolate salt (Scheme 1) or carbodiimide-mediated coupling, using the conditions described previously (Eq. 2). Formation of **42** required Boc-protection of N-phenylglycine [19] before DIC esterification with subsequent BOC removal after esterification. The planned analog **48** was to be a 2-pyridylphenoxyacetic acid ester of LNG, but spectroscopic analysis indicated the N-linked amide.

**Table 2:** LNG esters without sulfonamide function.

Compound	R	Method used^a^	Compound	R	Method used^a^
**22**		2	**36**		2
**23**		2	**37**		2
**24**		2	**38**		2
**25**		2	**39**		2
**26**		2	**40**		1
**27**		2	**41**		1
**28**		2	**42**		2
**29**		2	**43**		2
**30**		2	**45**		1
**31**		2	**46**		1
**32**		2	**47**		1
**33**		2	**48**		1
**34**		2	**49**		2
**35**		2			

a Method 1 – Displacement of Cl from intermediate 2 (see Scheme 1) Method 2 – Carbodiimide mediated esterification (see Eq. 2).

### 3.2 Formulation of analogs used in anti-ovulation study

Compounds in a crystalline form (or amorphous when crystals were unable to be attained) were suspended in a buffered formula (40 mg in 1.5 ml) as described in Bialy et al. [10] with the omission of polysorbate and then subjected to mechanical milling using 10 mm stainless steel ball bearings at a rate of 20 Hz for twenty minutes.

### 3.3 Biology

S-D rats (180–200 g) were observed for ten days prior to dosage (to ensure regular estrous cycling), then subjected to single sub-cutaneous injection. Estrus suppression was monitored by taking vaginal swabs every three days during a five day period (followed by two days of no observations, then a return to every three days during a five day period) and observing the epithelial and lamina propria microscopically (Figure 1); smears which showed cornified tissue indicated a return to an estrus and/or ovulating state at which time the activity was noted as ceased.

From the data listed in Table 3, several aspects are made evident; all of the sulfamoyl-amino-acid esters of LNG have arguably very poor anti-ovulatory activity in this assay. This trend was also observed with the non-amino sulfamoyl esters of LNG. Interestingly, the sodium salt **20** did give 14 ± 1.2 days of activity at the high dose but was deemed insignificant as MPA at this dose was significantly more effective. Also of slight interest was the mild activity at 4 mg of 3-enol-ester analog **5**. Meta sulfamoylphenoxyacetic acid analog **3** was clearly the most effective compound of this series. Curiously, this was the only analog of the series that was able to be crystallized effectively, although when tested head to head with its amorphous form there was not much difference in duration of ovulation suppression. The next series of compounds were based on analog **3** and set out to answer two questions- how important was the sulfonamide function and would other substitutions on the phenoxy ring result in better activity than **3**.

**Table 3:** Results from anti-ovulation assay of first series of compounds (average of 4 or 5 animals per group).

Compound	Structure	Rat anti-ovulatory activity (days)		Compound	Structure	Rat anti-ovulatory activity (days)	
		2 mg	4 mg			2 mg	4 mg
**3**		21 ± 7.0	34 + 3.2	**16**			1
**5**		1	7 ± 3.8	**17**			1
**11**		1	1	**18**			1
**12**			1	**19**			1
**13**			1	**20**		1	14 ± 1.2
**14**			1	**MPA (pos control)**		32 ± 8.3	40 ± 8.8
**15**			1				

From this data (Table 4) it is clear that compounds **22** and **38** were the frontrunners in the series. A quick glance at the data indicates the necessity for the phenoxyacetic acid moiety for superior anti-ovulation activity. Although some of the substituted phenoxyacetic acid derivatives are far less effective with regards to preventing ovulation than **22** and **38**, none of the compounds lacking the oxygen atom (**23**, **24**, **39**) give comparable anti-ovulation activity to these two leads. The differences in anti-ovulation activity regarding the substituted phenoxyacetic acids can be loosely described as such- in the case of methyl, methoxy, and chloro substituents, ortho substitution resulted in the fewest days of ovulation inhibition, in contrast with ortho-fluorine substitution resulting in the longest interval of inhibition for the fluorine substituted series. Fluorine substitution resulted in the highest additive number of days of inhibition at the 2 mg dose, while methoxy substitution resulted in the least number of days. Para-sulfonamide analog 4 inhibited ovulation twice as long as meta analog **3**. 3-pyridyl analog **37** inhibited ovulation over 50% less effectively than **22**.

**Table 4:** Results from anti-ovulation assay of second series of compounds.

Compound	Structure	Rat anti-ovulatory activity (days)		Compound	Structure	Rat anti-ovulatory activity (days) 2 mg
		2 mg	4 mg			
**4**		20 ± 6.2		**31**		30 ± 1.9
**22**		43 ± 5.4	69 ± 12.2	**32**		18 ± 1.7
**23**			20 ± 4.3	**33**		16 ± 5.2
**24**			6 ± 1.9	**34**		8 ± 0
**25**			36 ± 3.4	**35**		31 ± 6.6
**26**			45 ± 6.1	**36**		31 ± 5.9
**27**			47 ± 3.0	**37**		23 ± 1.0
**28**		34 ± 3.2		**38**		41 ± 3.6
**29**		8 ± 0.6		**39**		3 ± 0.8
**30**		16 ± 5.4				

Also of interest is the comparison between analog **22** and its α-methyl relative **34**. It is possible that this methyl group inhibits ester hydrolysis to the active compound, and the prodrug is eliminated intact.

Our next series of compounds explored SAR of the aromatic function in addition to other heteroatoms linking the carboxyl group to the aromatic function besides oxygen, as well as the synthesis and determining the anti-ovulatory activity of the phenoxyacetic acid ester of ENG.

Analysis of this raw data (Table 5) make clear two concepts-none of these analogs prevent ovulation in rats for as long as the phenoxyacetic acid analog **22**, and that placing this group on ENG (analog **44**) gave a compound which gave the longest duration of anti-ovulatory activity tested to date. Of interest, ENG has a markedly reduced duration of anti-ovulatory activity in the assay compared to LNG (8 + 2.1 vs 28 + 0.7 days at the 2 mg dose).

**Table 5:** Results from anti-ovulation assay of third series of compounds.

Compound	Structure	Rat anti-ovulatory activity (days), 2 mg dose
**40**		20 ± 3.7
**41**		31 ± 7.4
**42**		13 ± 0.5
**43**		20 ± 3.1
**44**		60 ± 6.9
**45**		27 ± 1.5
**46**		8 ± 0.8
**47**		37 ± 1.0
**48**		5 ± 1.0
**49**		29 ± 4.7

Changing the aromatic group while maintaining the methylene linker between acyl and aryl groups did not result in any activity better than **22** or **44**. Also, changing the aryl-linked heteroatom did not result in any improvement of activity, although somewhat interestingly chalcogen **43** had markedly better activity than pnictogen **42**. Also of interest is the much longer duration of anti-ovulation activity in para-biphenyl analog **47** vs meta **46**. This is somewhat akin to what was observed in the second series of compounds, with para substitution giving the superior activity, with the exception of the fluorine substituents.

## 4 Discussion

Analysis of this raw anti-ovulatory activity data seems to suggest a rather limited number of these ester pro-drugs could be considered as truly superior in causing cessation of ovulation in rats. What seems to be very basic requirements for what could be considered the most effective contraceptives of this group is the presence of an alpha-oxygen atom, one methylene unit between the ester carbonyl function and the oxygen atom, and an aromatic moiety bound to the oxygen atom that is not a heterocycle. This rather “tight” SAR was also noted in previous work regarding esters of LNG by Bialy et al. [10] For instance, one of the most effective compounds noted by these workers was levonorgestrel butyrate (LB, inhibited ovulation for 34 ± 3 days at 2 mg). By comparison, changing the number of carbon atoms in the chain by just one unit in either direction resulted in substantially lower number of days of activity at all doses studied (propanoate ester inhibited ovulation for 17 ± 1 days at 2 mg and pentanoate inhibited ovulation for 14 ± 2 days at 2 mg). Ringed aliphatic esters cyclopropanoate and cyclobutanoate gave far greater activity (37 ± 2 days and 32 days both at 2 mg) than cyclopentanoate and cyclohexanoate (4 ± 1 day and 7 ± 3 days both at 2 mg), again illustrating the small window of activity among similar analogs.

Trying to rationalize the overall activity using single parameters when comparing the compounds does not result in any noticeable trends or plausible explanations. Take for instance the idea that higher melting compounds result in a more long-lasting depot (as more time and biological media would be required to disrupt the crystal lattice in theory). This idea is easily nullified by comparing **22** and **44**; **44** melts at 160 °C while **22** melts at 206 °C, yet **44** gives the longest activity of all analogs. Compound **42** was one of the highest melting compounds listed in this article (243 °C), but exhibits poor activity in comparison.

Another concept taken into account is ester hydrolysis. It was noted by Bialy et al. that some esters with very slow hydrolysis rates still resulted in activity. It was not mentioned whether or not these compounds were tested for progesterone receptor binding.

As speculation regarding explanation of activity of these analogs could be extensive, a reasonable way to explain these observations is a “perfect storm” of all parameters-ester hydrolysis, crystallinity, aqueous solubility, particle size, and perhaps most importantly, formulation, must form and additively contribute to optimal anti-ovulation activity. It does indeed seem that compounds **22** and **44** meet all these criteria, as these (especially **44**) have been found to have nearly double the anti-ovulatory activity of MPA in the rat model. Challenges exist to get these compounds into the clinic for human use. Besides the obvious issues of toxicity (in this case, the parent is an approved drug, however any intact pro-drug which did not hydrolyze and the pro-drug piece itself would have to be subjected to tox studies), one issue that we believe to be crucial is human bioavailability. This concern would stem from the case of LB mentioned earlier. In the 1970’s, this was arguably the WHO’s most promising compound (again, in rats) as a single-injectable, long-term contraceptive, and has undergone human clinical trials up to phase 3, but to this date has not been either approved or marketed. It is unclear why this might be the case, but one cause could possibly be inconsistencies in duration of anti-ovulation from individual subject to subject. It is quite possible that these inconsistencies (assuming this to be the case) are caused by a deficiency in the formulation. Now that particle sizing technologies have progressed well beyond the 1970’s, our group has been focusing on using jet-milling technology to produce spherical, tightly distributed particle sizes of each prodrug. The tight distributions will have small (Dv 50 of 5 µm), medium (Dv 50 of 12 µm), and large (Dv 50 of 18 µm) average sized particles. Each particle set will then be tested for anti-ovulation activity in the rat model, to determine whether an optimal Dv 50 gives even longer duration of anti-ovulation (compared to previous batches which had wider size-distribution). Also, we are studying the injection volume of the formulated pro-drugs, to determine if this plays a role in anti-ovulation duration. These studies along with other topics such as polymorphism and ester hydrolysis studies are to be described in future articles.

## 5 Summary

A series of compounds were made using a hypothesis based on previous work involving steroidal human carbonic anhydrase ligands for improved bioavailability, and when probed for anti-ovulation activity in a murine model identified only one compound with satisfactory activity. Subsequent analogs based loosely on this single initial compound yielded phenoxyacetic acid esters of LNG and ENG which resulted in anti-ovulation activity of substantially longer duration in comparison to MPA, and are currently undergoing further studies.

## References

[1] United Nations (UN), Department of Economic and Social Affairs, Population Division. World contraceptive use 2011. New York: UN; 2011. Available from: http://www.un.org/esa/population/publications/contraceptive2011/contraceptive2011.htm.

[2] McKenna K., Arcara J., Rademacher K., Mackenzie C., Ngabo F., Munyambanza E., Wesson J., Tolley E. (2014). Policy and programmatic considerations for introducing a longer-acting injectable contraceptive: perspectives of stakeholders from Kenya and Rwanda. Global Health: Sci. Practice.

[3] Trussell J., Hatcher R.A., Trussell J., Stewart F.H., Nelson A.L., Cates W., Guest F., Kowal D. (2004). Contraceptive efficacy. Contraceptive Technology (18th rev. ed.).

[4] Trussell J. (2004). Contraceptive failure in the United States. Contraception.

[5] http://www.pdr.net/drug-summary/Depo-Provera-Contraceptive-Injection-medroxyprogesterone-acetate-1373.

[6] Westhoff C. (2003). Depot-medroxyprogesterone acetate injection (Depo-Provera®): a highly effective contraceptive option with proven long-term safety. Contraception.

[7] https://web.archive.org/web/20051221195621/http://www.fda.gov/bbs/topics/ANSWERS/2004/ANS01325.html.

[8] Rosseow J.E., Anderson G.L., Prentice R.L., LeCroix A.Z., Kooperberg C., Stefanick M.L., Jackson R.D., Beresford S.A., Howard B.V., Johnson K.C., Kotchen J.M., Ockene J. (2002). Risks and benefits of estrogen plus progestin in healthy postmenopausal women: principal results From the Women’s Health Initiative randomized controlled trial. JAMA.

[9] Shafiee A., Vossoghi M., Savabi F., Vlahov R., Tarpanov V., Boshkova-Ljapova M., Milenkov B., Stoilova V., Vlahov J., Snatzke G. (1983). Long-acting contraceptive agents: cyclopropyl and cyclobutyl esters of norethisterone. Steroids.

[10] Bialy G., Blye R.P., Enever R.P., Naqvi R.H., Lindberg M.C. (1983). Long-acting contraceptive agents: Structure activity relationships in a series of norethisterone and levonorgestrel esters. Steroids.

[11] Crabbé P., Archer S., Benagiano G., Diczfalusy E., Djerassi C., Fried J., Higuchi T. (1983). Long-acting contraceptive agents: design of the WHO chemical synthesis programme. Steroids.

[12] Benagiano G., d’Arcangues C., Harris R., Schafer A., Say L., Merialdi M. (2012). The special programme of research in human reproduction: forty years of activities to archive reproductive health for all. Gynecol. Obstet. Invest..

[13] Edelman A.B., Cherala G., Li H., Blithe D.L., Jensen J.T. (2017). Levonorgestrel butanoate intramuscular injection does not reliably suppress ovulation for 90 days in obese and normal-BMI women: a pilot study. Contraception.

[14] Ahmed G., Elger W., Meece F., Nair H., Schneider B., Wyrwa R., Nickisch K. (2017). A prodrug design for improved oral absorption and reduced hepatic interaction. Bioorg. Med. Chem..

[15] S.P. Sahoo, H. Koyama, D.J. Miller, N-cyclohexylaminocarbonylbenzenesulfoamide derivatives WO 2004066963.

[16] Herz J.E., Leslie Gunatilaka A.A., Sotheeswaran S., Torres J.V. (1982). Esterification of acid chlorides with thallium and potassium salts of 19-norethisterone: formation of 17-enol esters. Steroids.

[17] Leung S.L., Karunanity R., Becket G., Yeo S.H. (1985). Norethisterone and levonorgestrel esters: a novel synthetic method. Steroids.

[18] Reuillon T., Bertoli A., Griffin R.J., Miller D.C., Golding B.T. (2012). Efficacious N-protection of O-aryl sulfamates with 2,4-dimethoxybenzyl groups. Org. Biomol. Chem..

[19] Lee W.S., Lee M.J., Kim B.J., Roh T.C., Lee S.H., Lee K.D., You-Hui K., Kwak T.H. (2015). 1,2-Napthoquinone derivative and method for preparing same. Pct. Int. Appl..

