# Supplementary material for: Esters of levonorgestrel and etonogestrel intended as single, subcutaneous-injection, long-lasting contraceptives
Source: Steroids. 2018 Sep;137:47–56. doi: 10.1016/j.steroids.2018.07.010 (PMC6137153; doi:10.1016/j.steroids.2018.07.010)
Supplement: Supplementary data 1 [file mmc1.docx]

Supporting Information

Esters of Levonorgestrel and Etonogestrel Intended as Single-Injection, Long-Lasting Contraceptives

Frederick A Meece, Gulzar Ahmed, Hareesh Nair, Bindu Santhamma, Rajeshwar R Tekmal, Chumang Zhao, Nicole Pollok, Julia Lara, Ze’ev Shaked, Klaus Nickisch

Content- Spectroscopic data for compounds not given in main body

List of Abbreviations

DCM- dichloromethane

DIC- N,N’-diisopropylcarbodiimide

DIEA- diisopropylethylamine

DMAP- 4-dimethylaminopyridine

DMF- dimethylformamide

EtOAc- ethyl acetate

HOBt- 1-hydroxybenzotriazole hydrate

Hz- hertz

MeOH- methanol

TEA- triethylamine

TFA- trifluoroacetic acid

THF- tetrahydrofuran

Spectral Data

**2** (1R,11aS)-11a-ethyl-1-ethynyl-7-oxohexadecahydro-1H-cyclopenta[a]phenanthren-1-yl chloroacetate; ^1^H NMR (300 MHz, CDCl_3_) δ 5.82 (s, 1H), 4.02 (s, 2H), 2.65 (s, 1H).

**4** (1R,11aS)-11a-ethyl-1-ethynyl-7-oxo-2,3,3a,3b,4,5,7,8,9,9a,9b,10,11,11a-tetradecahydro-1H-cyclopenta[a]phenanthren-1-yl (4-sulfamoylphenoxy)acetate; ^1^H NMR (CDCl_3,_ 300 MHz) δ 7.87 (d, 2H, *J* = 9.0 Hz), 6.96 (d, 2H, *J* = 9.0 Hz), 5.83 (s, 1H), 4.77 (s, 2H), 4.67 (s, 2H), 2.67 (s, 1H), 0.97 (t, 3H, CH3, *J* = 7.2 Hz). IR (cm^-1^): 3259, 2939, 2875, 1760, 1704, 1651. MP 115 – 117^o^C.

**19** (13S,17R)-13-ethyl-17-ethynyl-3-oxo-2,3,6,7,8,9,10,11,12,13,14,15,16,17-tetradecahydro-1H-cyclopenta[a]phenanthren-17-yl 3-sulfamoylpropanoate; ^1^H NMR (300 MHz, DMSO-d_6_) δ 6.91 (s, 2H), 5.73 (s, 1H), 3.59 (s, 1H), 3.22 (t, *J* = 7.8 Hz, 2H), 2.72 (t, *J* = 7.5 Hz, 2H). IR (cm^-1^): 3264, 2954, 2906, 2866, 2839, 1739, 1651, 1462, 1366, 1145. MP 141 – 144^o^C.

**22** (1R,11aS)-11a-ethyl-1-ethynyl-7-oxo-2,3,3a,3b,4,5,7,8,9,9a,9b,10,11,11a-tetradecahydro-1H-cyclopenta[a]phenanthren-1-yl phenoxyacetate; ^1^H NMR (CDCl_3_, 300 MHz) δ 7.29 (dt, 2H, *J* = 6.6, 0.9 Hz), 7.00 (dt, 1H, *J* = 7.0, 0.9 Hz), 6.89 (dd, 2H, *J* = 8.1, 0.9 Hz), 5.84 (s, 1H), 4.61 (s, 2H), 2.82 (m, 2H), 2.65 (s, 1H) 0.99 (t, 3H, *J* = 7.2 Hz). ^13^C NMR (CDCl_3_, 75MHz) δ 199.7, 167.6, 166.2, 157.7, 129.5, 124.7, 121.6, 114.5, 87.2, 82.6, 75.9, 65.5, 49.0, 48.8, 48.7, 42.3, 40.6, 37.6, 36.5, 35.4, 30.6, 28.5, 26.5, 26.1, 22.9, 19.6, 9.3. IR (cm^-1^): 3225, 2939, 2873, 1775, 1745, 1658. MP 202.4 – 203.3^o^C.

**23** (1R,11aS)-11a-ethyl-1-ethynyl-7-oxo-2,3,3a,3b,4,5,7,8,9,9a,9b,10,11,11a-tetradecahydro-1H-cyclopenta[a]phenanthren-1-yl 3-phenylpropanoate; ^1^H NMR (CDCl_3_, 300 MHz) δ 7.24 (m, 5H), 5.83 (s, 1H), 2.95 (t, 2H, *J* = 7.9 Hz), 2.60 (s, 1H) 0.99 (t, 3H, *J* = 7.3 Hz). ^13^C NMR (δ, CDCl_3_ 75 MHz): 199.7, 171.4, 166.2, 140.3, 128.4, 128.3, 126.2, 124.6, 85.8, 83.3, 75.1, 75.0, 48.88, 48.83, 48.70, 42.3, 40.6, 37.7, 36.48, 36.40, 35.4, 30.9, 30.6, 28.5, 26.5, 26.1, 22.8, 19.5, 9.3. IR (cm^-1^): 3227, 2938, 2872, 1742, 1659. MP 183 – 184^o^C.

**25** (13S,17R)-13-ethyl-17-ethynyl-3-oxo-2,3,6,7,8,9,10,11,12,13,14,15,16,17-tetradecahydro-1H-cyclopenta[a]phenanthren-17-yl (3-methylphenoxy)acetate; ^1^H NMR (300 MHz, CDCl_3_) δ 7.17 (t, *J* = 7.5 Hz, 1H), 6.81 (d, *J* = 7.2 Hz, 1H), 6.73 – 6.67 (m, 2H), 5.84 (s, 1H), 4.59 (s, 2H), 2.66 (s, 1H), 2.33 (s, 3H). IR (cm^-1^): 3227, 1775, 1658, 1611, 1259, 1196, 1158, 1095, 785, 688. MP 187 – 190^o^C.

**26** (13S,17R)-13-ethyl-17-ethynyl-3-oxo-2,3,6,7,8,9,10,11,12,13,14,15,16,17-tetradecahydro-1H-cyclopenta[a]phenanthren-17-yl (4-methylphenoxy)acetate; ^1^H NMR (300 MHz, CDCl_3_) δ 7.08 (d, *J* = 8.4 Hz, 2H), 6.79 (d, *J* = 8.7 Hz, 2H), 5.84 (s, 1H), 4.57 (s, 2H), 2.66 (s, 1H), 2.29 (s, 3H). IR (cm^-1^): 3251, 1746, 1658, 1510, 1220, 680. MP 177 – 180^o^C.

**27** (13S,17R)-13-ethyl-17-ethynyl-3-oxo-2,3,6,7,8,9,10,11,12,13,14,15,16,17-tetradecahydro-1H-cyclopenta[a]phenanthren-17-yl (3-chlorophenoxy)acetate; ^1^H NMR (300 MHz, CDCl_3_) δ 7.22 (t, *J* = 8.1 Hz, 1H), 6.99 (dq, *J* = 8.1, 0.9 Hz, 1H), 6.88 (t, *J* = 2.1 Hz, 1H), 6.80 (ddd, *J* = 8.1, 2.4, 0.9 Hz, 1H), 5.85 (s, 1H), 4.60 (s, 2H), 2.68 (s, 1H). IR (cm^-1^): 3224, 1780, 1658, 1591, 1091, 862, 788, 680. MP 170 – 173^o^C.

**28** (13S,17R)-13-ethyl-17-ethynyl-3-oxo-2,3,6,7,8,9,10,11,12,13,14,15,16,17-tetradecahydro-1H-cyclopenta[a]phenanthren-17-yl (4-chlorophenoxy)acetate; ^1^H NMR (300 MHz, CDCl_3_) δ 7.27 – 7.22 (m, 2H), 6.85 – 6.81 (m, 2H), 5.85 (s, 1H), 4.58 (s, 2H), 2.67 (s, 1H). IR (cm^-1^): 3224, [1780, 1748 Fermi doublet], 1658, 1489, 1199, 1071, 828, 700. MP 169 – 172^o^C.

**29** (13S,17R)-13-ethyl-17-ethynyl-3-oxo-2,3,6,7,8,9,10,11,12,13,14,15,16,17-tetradecahydro-1H-cyclopenta[a]phenanthren-17-yl (2-methylphenoxy)acetate; ^1^H NMR (300 MHz, CDCl_3_) δ 7.17 – 7.11 (m, 2H), 6.91 (t, *J* = 7.2 Hz, 1H), 6.69 (d, *J* = 8.1 Hz, 1H), 5.84 (s, 1H), 4.63 (s, 2H), 2.66 (s, 1H), 2.28 (s, 3H). IR (cm^-1^): 3224, 2103, 1780, 1658, 1193, 1125, 1071, 761, 700. MP 189.5 – 193.5^o^C.

**30** (13S,17R)-13-ethyl-17-ethynyl-3-oxo-2,3,6,7,8,9,10,11,12,13,14,15,16,17-tetradecahydro-1H-cyclopenta[a]phenanthren-17-yl (2-chlorophenoxy)acetate; ^1^H NMR (300 MHz, CDCl_3_) δ 7.39 (dd, *J* = 7.8, 1.5 Hz, 1H), 7.25 – 7.17 (m, 1H), 6.96 (td, *J* = 7.8, 1.5 Hz, 1H), 6.84 (dd, *J* = 8.4, 0.9 Hz, 1H), 5.84 (s, 1H), 4.70 (s, 2H), 2.66 (s, 1H). IR (cm^-1^): 3227, 2103, 1771, 1658, 1612, 1477, 1196, 1070, 1053, 772, 701. MP 194.5 – 198^o^C.

**31** (13S,17R)-13-ethyl-17-ethynyl-3-oxo-2,3,6,7,8,9,10,11,12,13,14,15,16,17-tetradecahydro-1H-cyclopenta[a]phenanthren-17-yl (4-methoxyphenoxy)acetate; ^1^H NMR (300 MHz, CDCl_3_) δ 6.84 (s, 4H), 5.84 (s, 1H), 4.56 (s, 2H), 3.78 (s, 3H), 2.66 (s, 1H). IR (cm^-1^): 3311, 1763, 1660, 1612, 1503, 1165, 1085, 1031, 820. MP 126.5 – 130^o^C.

**32** (13S,17R)-13-ethyl-17-ethynyl-3-oxo-2,3,6,7,8,9,10,11,12,13,14,15,16,17-tetradecahydro-1H-cyclopenta[a]phenanthren-17-yl (2-methoxyphenoxy)acetate; ^1^H NMR (300 MHz, CDCl_3_) δ 7.04 – 6.75 (m, 4H), 5.84 (s, 1H), 4.68 (s, 2H), 3.88 (s, 3H), 2.65 (s, 1H). IR (cm^-1^): 3257, 2210, 1773, 1655, 1500, 1186, 1085, 1071, 761. MP 196 – 199^o^C.

**33** (13S,17R)-13-ethyl-17-ethynyl-3-oxo-2,3,6,7,8,9,10,11,12,13,14,15,16,17-tetradecahydro-1H-cyclopenta[a]phenanthren-17-yl (3-methoxyphenoxy)acetate; ^1^H NMR (300 MHz, CDCl_3_) δ 7.22 – 7.16 (m, 1H), 6.56 (ddd, *J* = 8.1, 3.0, 0.9 Hz, 1H), 6.49 – 6.45 (m, 2H), 5.84 (s, 1H), 4.59 (s, 2H), 3.80 (s, 3H), 2.66 (s, 1H). IR (cm^-1^): 3224, 2210, 1773, 1655, 1611, 1193, 855, 774. MP 156 – 159^o^C.

**37** (13S,17R)-13-ethyl-17-ethynyl-3-oxo-2,3,6,7,8,9,10,11,12,13,14,15,16,17-tetradecahydro-1H-cyclopenta[a]phenanthren-17-yl [(pyridin-3-yl)oxy]acetate; ^1^H NMR (300 MHz, CDCl_3_) δ 8.33 – 8.27 (m, 2H), 7.30 – 7.18 (m, 2H), 5.85 (s, 1H), 4.66 (s, 2H), 2.68 (1H). IR (cm^-1^): 3223, 2103, 1775, 1658, 1427, 1262, 1196, 1083, 801, 705. MP 167 – 170.5^o^C.

**38** (1R,11aS)-11a-ethyl-1-ethynyl-7-oxo-2,3,3a,3b,4,5,7,8,9,9a,9b,10,11,11a-tetradecahydro-1H-cyclopenta[a]phenanthren-1-yl (2-fluorophenoxy)acetate; ^1^H NMR (CDCl_3_, 300 MHz) δ 6.98 (m, 4H), 5.82 (s, 1H), 4.66 (s, 2H), 2.81 (m, 1H), 2.64 (s, 1H) 0.94 (t, *J* = 7.3 Hz). IR (cm^-1^): 3225, 2938, 2870, 1775, 1660. MP 185 – 186^o^C.

**40** (1R,11aS)-11a-ethyl-1-ethynyl-7-oxo-2,3,3a,3b,4,5,7,8,9,9a,9b,10,11,11a-tetradecahydro-1H-cyclopenta[a]phenanthren-1-yl [(2H-1,3-benzodioxol-5-yl)oxy]acetate; ^1^H NMR (CDCl_3_, 300 MHz) δ 6.69 (d, 1H, *J* = 7.8 Hz), 6.51 (d, 1H, *J* = 2.7 Hz), 6.29 (dd, 1H, *J* = 2.7, 8.7 Hz), 5.93 (s, 2H), 5.84 (s, 1H), 4.52 (s, 2H), 2.82 (m, 1H), 2.65 (s, 1H) 1.00 (t, 3H, *J* = 7.0 Hz). IR (cm^-1^): 3222, 2941, 2872, 1772, 1659. MP 166 – 166.5^o^C.

**41** (1R,11aS)-11a-ethyl-1-ethynyl-7-oxo-2,3,3a,3b,4,5,7,8,9,9a,9b,10,11,11a-tetradecahydro-1H-cyclopenta[a]phenanthren-1-yl [4-(methanesulfonyl)phenoxy]acetate; ^1^H NMR (CDCl_3_, 300 MHz) δ 7.88 (dd, 2H, *J* = 2.1, 6.9 Hz), 7.01 (dd, 2H, *J* = 2.1, 6.9 Hz), 5.84 (s, 1H), 4.69 (s, 3H), 3.03 (s, 2H), 2.80 (m, 1H), 2.68 (s, 1H) 0.97 (m, 3H). IR (cm^-1^): 3264, 2938, 2873, 1763, 1663. MP 198.4 – 199.1^o^C.

**42** (13S,17R)-13-ethyl-17-ethynyl-3-oxo-2,3,6,7,8,9,10,11,12,13,14,15,16,17-tetradecahydro-1H-cyclopenta[a]phenanthren-17-yl anilinoacetate; ^1^H NMR (300 MHz, CDCl_3_) δ 7.19 (td, *J* = 7.5, 2.1 Hz, 2H), 6.76 (t, *J* = 7.5 Hz, 1H), 6.61 (dd, *J* = 8.7, 1.2 Hz, 2H), 5.84 (s, 1H), 4.28 (bs, 1H), 3.89 (d, *J* = 3.6 Hz, 2H), 2.65 (s, 1H). IR (cm^-1^): 3408, 3223, 1750, 1658, 1603, 1511, 1360, 1259, 1200, 755, 692. MP 234 – 237.5^o^C.

**43** (13S,17R)-13-ethyl-17-ethynyl-3-oxo-2,3,6,7,8,9,10,11,12,13,14,15,16,17-tetradecahydro-1H-cyclopenta[a]phenanthren-17-yl (phenylsulfanyl)acetate; ^1^H NMR (300 MHz, CDCl_3_) δ 7.45 – 7.40 (m, 2H), 7.34 – 7.20 (m, 3H), 5.84 (s, 1H), 3.61 (s, 2H), 2.60 (1H). IR (cm^-1^): 3227, 1742, 1653, 1603, 1263, 1116, 1008, 743, 688. MP 165.5 – 168^o^C.

**44** (1R,11aS)-11a-ethyl-1-ethynyl-10-methylidene-7-oxo-2,3,3a,3b,4,5,7,8,9,9a,9b,10,11,11a-tetradecahydro-1H-cyclopenta[a]phenanthren-1-yl phenoxyacetate; ^1^H NMR (CDCl_3_, 300 MHz) δ 7.29 (td, 2H, *J* = 7.5, 2.1 Hz), 6.99 (tt, 1H, *J* = 7.2, 0.9 Hz), 6.89 (dd, 2H, *J* = 7.5, 0.9 Hz), 5.89 (s, 1H), 5.06 (s, 1H), 4.85 (s, 1H), 4.61 (s, 2H), 2.86 (m, 2H), 2.69 (s, 1H) 1.03 (t, 3H, *J* = 7.2 Hz). ^13^C NMR (δ, CDCl_3_ 75 MHz): 199.8, 167.4, 166.0, 157.5, 145.8, 129.4, 125.6, 121.6, 114.4, 109.3, 86.3, 82.4, 76.0, 65.4, 53.4, 51.1, 50.1, 41.5, 40.5, 37.8, 37.4, 36.8, 35.1, 30.0, 28.1, 22.1, 20.3, 8.7. IR (cm^-1^): 3291, 3217, 1766, 1658, 1489, 1190, 1085, 902, 761, 693. MP 159.7 – 161.6^o^C.

**45** (1R,11aS)-11a-ethyl-1-ethynyl-7-oxo-2,3,3a,3b,4,5,7,8,9,9a,9b,10,11,11a-tetradecahydro-1H-cyclopenta[a]phenanthren-1-yl [(5-methyl-1,2-oxazol-3-yl)oxy]acetate; ^1^H NMR (CDCl_3_, 300 MHz) δ 5.83 (s, 1H), 5.70 (d, 1H, *J* = 0.6 Hz), 4.76 (s, 2H), 2.84 (m, 1H), 2.65 (s, 1H), 2.33 (t, 3H, *J* = 0.6 Hz), 0.98 (t, 3H, *J* = 7.3 Hz) IR (cm^-1^): 3263, 3139, 2947, 2878, 1764, 1668. MP 165.7 – 166.1^o^C.

**46** (13S,17R)-13-ethyl-17-ethynyl-3-oxo-2,3,6,7,8,9,10,11,12,13,14,15,16,17-tetradecahydro-1H-cyclopenta[a]phenanthren-17-yl [([1,1'-biphenyl]-3-yl)oxy]acetate; ^1^H NMR (300 MHz, CDCl_3_) δ 7.65 – 7.50 (m, 2H), 7.47 – 7.31 (m, 3H), 7.30 – 7.20 (m, 2H), 7.11 (t, *J* = 1.8 Hz, 1H), 6.87 (ddd, *J* = 7.8, 2.4, 0.6 Hz, 1H), 5.83 (s, 1H), 4.67 (s, 2H), 2.61 (s, 1H). IR (cm^-1^): 3278, 1767, 1742, 1666, 1473, 1255, 1167, 1078, 978, 860, 766, 692, 608.

**47** (13S,17R)-13-ethyl-17-ethynyl-3-oxo-2,3,6,7,8,9,10,11,12,13,14,15,16,17-tetradecahydro-1H-cyclopenta[a]phenanthren-17-yl [([1,1'-biphenyl]-4-yl)oxy]acetate. ^1^H NMR (300 MHz, CDCl3) δ 7.60 – 7.49 (m, 3H), 7.47 – 7.37 (m, 2H), 7.35 – 7.26 (m, 2H), 6.99 – 6.94 (m, 2H), 5.83 (s, 1H), 4.65 (s, 2H), 2.67 (s, 1H). IR (cm^-1^): 3284, 1766, 1651, 1179, 754.

**48** (13S,17R)-13-ethyl-17-ethynyl-3-oxo-2,3,6,7,8,9,10,11,12,13,14,15,16,17-tetradecahydro-1H-cyclopenta[a]phenanthren-17-yl (2-oxopyridin-1(2H)-yl)acetate. ^1^H NMR (300 MHz, DMSO-d_6_) δ 7.67 (dd, *J* = 6.6, 1.5 Hz, 1H), 7.46 (tddd, *J* = 15.9, 11.4, 9.0, 2.1 Hz, 1H), 6.41 (d, *J* = 9.0 Hz, 1H), 6.25 (td, *J* = 13.5, 6.6, 1.5 Hz, 1H) 5.72 (s, 1H), 4.64 (s, 1H), 3.63 (s, 1H). IR (cm^-1^): 3294, 1750, 1679, 1662, 1586, 1536, 1355, 1251, 1238, 1196, 1007, 768, 680, 642. MP 171 – 175^o^C.

**49** (1R,11aS)-11a-ethyl-1-ethynyl-7-oxo-2,3,3a,3b,4,5,7,8,9,9a,9b,10,11,11a-tetradecahydro-1H-cyclopenta[a]phenanthren-1-yl (4-cyanophenoxy)acetate. ^1^H NMR (CDCl_3_, 300 MHz) δ 7.99 (dd, 2H, *J* = 2.1, 9.6 Hz), 6.93 (dd, 2H, *J* = 2.1, 9.0 Hz), 5.82 (s, 1H), 4.65 (s, 2H), 2.79 (m, 1H), 2.66 (s, 1H) 0.95 (t, 3H, *J* = 7.2 Hz). IR (cm^-1^): 3277, 2938, 2875, 2223, 1763, 1664. MP 92 – 93^o^C.
